# Prenatal prediction of neonatal haemodynamic adaptation after maternal hyperoxygenation

**DOI:** 10.1186/s12884-020-03403-y

**Published:** 2020-11-19

**Authors:** Ann McHugh, Colm Breatnach, Neidin Bussmann, Orla Franklin, Afif El-Khuffash, Fionnuala M. Breathnach

**Affiliations:** 1 Department of Obstetrics and Gynaecology, Royal College of Surgeons in Ireland, Rotunda Hospital, Dublin, Ireland; 2 Department of Neonatology, Royal College of Surgeons in Ireland, Rotunda Hospital, Dublin, Ireland; 3 Children’s Health Ireland at Crumlin, Dublin, Ireland

**Keywords:** Hyperoxygenation, Doppler echocardiography, Pulmonary artery Doppler

## Abstract

**Abstract:**

The reactivity of the pulmonary vascular bed to the administration of oxygen is well established in the post-natal circulation. The vasoreactivity demonstrated by the fetal pulmonary artery Doppler waveform in response to maternal hyperoxia has been investigated. We sought to investigate the relationship between the reactivity of the fetal pulmonary arteries to hyperoxia and subsequent neonatal cardiac function in the early newborn period.

**Methods:**

This explorative study with convenience sampling measured pulsatility index (PI), resistance index (RI), acceleration time (AT), and ejection time (ET) from the fetal distal branch pulmonary artery (PA) at baseline and following maternal hyperoxygenation (MH). Oxygen was administered for 10 min at a rate of 12 L/min via a partial non-rebreather mask. A neonatal functional echocardiogram was performed within the first 24 h of life to assess ejection fraction (EF), left ventricular output (LVO), and neonatal pulmonary artery AT (nPAAT). This study was conducted in the Rotunda Hospital, Dublin, Ireland.

**Results:**

Forty-six women with a singleton pregnancy greater than or equal to 31 weeks’ gestational age were prospectively recruited to the study. The median gestational age was 35 weeks. There was a decrease in fetal PAPI and PARI following MH and an increase in fetal PAAT, leading to an increase in PA AT:ET. Fetuses that responded to hyperoxygenation were more likely to have a higher LVO (135 ± 25 mL/kg/min vs 111 ± 21 mL/kg/min, *p* < 0.01) and EF (54 ± 9% vs 47 ± 7%,*p* = 0.03) in the early newborn period than those that did not respond to MH prenatally. These findings were not dependent on left ventricular size or mitral valve (MV) annular diameter but were related to an increased MV inflow. There was no difference in nPAAT.

**Conclusion:**

These findings indicate a reduction in fetal pulmonary vascular resistance (PVR) and an increase in pulmonary blood flow and left atrial return following MH. The fetal response to hyperoxia reflected an optimal adaptation to postnatal life with rapid reduction in PVR increasing measured cardiac output.

**Supplementary Information:**

The online version contains supplementary material available at 10.1186/s12884-020-03403-y.

## Background

In utero, the placenta functions as the organ for gaseous exchange [1]. A high pulmonary vascular resistance (PVR) is a normal state for the fetus and pulmonary vascular tone increases with advancing gestational age (GA) [2]. Fetal pulmonary arterial vascular impedance decreases during the second half of pregnancy until 34 to 35 weeks GA [3]. Despite ongoing lung growth after 34 to 35 weeks’ gestation, the pulmonary vascular impedance thereafter remains unchanged [4]. At ≥37 weeks gestation, pulmonary blood flow increases substantially to almost half of the right ventricular output [5]. A low oxygen tension environment exists in utero, which promotes high intrinsic myogenic tone and high vasocontractility [6]. At birth, there is a reduction in pulmonary arterial pressure and resistance, due to an increase in oxygen tension and up to a ten-fold rise in pulmonary blood flow [6]. Neonatal survival is dependent upon a rapid, complex and well-orchestrated transition from the intra- to extrauterine environment [7]. Normal transition to newborn circulation requires high fetal pressures to fall, with dilatation of the pulmonary vessels. Previous studies have indicated that the capacity of pulmonary arteries to dilate can be judged prenatally, by administering high-dose oxygen to the mother [8–10].

### Hyperoxygenation

Studies have shown that fetal pulmonary vasculature reacts to maternal hyperoxygenation (MH) [4, 11, 12]. Oxygen induces the release of several vasodilators including endothelium-derived nitric oxide and prostacyclin, resulting in a decrease in PVR and an increase in pulmonary blood flow [13]. Following maternal oxygen administration, a decrease in the fetal PVR, as demonstrated by the pulmonary artery (PA) Doppler, is deemed to indicate vasoreactivity in the pulmonary vascular bed [14]. The reactivity of the PA to changes in fetal oxygen tension can be detected by noninvasive Doppler ultrasound techniques between 31 and 36 weeks GA [4]. Nomograms of pulmonary reactivity induced by hyperoxia during gestation have been established [13]. The measurement of pulmonary velocity waveforms before and after MH may therefore help in predicting how the fetus will adapt to the extra-uterine environment and transition to neonatal life. If there is a failure of the normal circulatory transition in the early newborn period, persistence of the fetal circulation occurs, resulting in pulmonary hypertension, low oxygen levels and may result in right-to-left shunting of blood in the newborn heart [2]. Some degree of pulmonary hypertension complicates the course of more than 10% of all neonates with respiratory failure [15]. Approximately 10% of neonates will require some form of clinical intervention at birth with 1% requiring more extensive resuscitation [16]. It is vitally important for clinicians to understand the changes that occur during the transition to neonatal life and to predict which neonates may have difficulty transitioning [16]. Prediction of neonatal pulmonary hypertension may influence the delivery planning for particularly high-risk cases and help guide the pharmacological and neonatal intensive care strategies that optimise postnatal survival. The ability to predict the fetal transition to neonatal life by a method that is non-invasive and reproducible would be beneficial for obstetric management, for parental counselling and for determining optimal neonatal management.

In this explorative prospective study, we hypothesise that the degree of the fetal pulmonary vascular bed response to maternal hyperoxia assessed using pulmonary artery Doppler ultrasound at ≥31 weeks’ gestation can predict transition during the early neonatal period.

### Study objective

To evaluate changes in the fetal pulmonary artery Doppler following maternal hyperoxygenation during the third trimester of pregnancy and to correlate those findings with neonatal echocardiograph indices indicative of increased pulmonary systolic pressures, including estimates of right ventricular systolic pressures, PA acceleration time in addition to measurements of neonatal ejection fraction and left ventricular output.

## Methods

This explorative prospective pilot study was undertaken in the Department of Obstetrics and Gynaecology in the Rotunda Hospital Dublin, Ireland between January 2017 and July 2018. The Rotunda Hospital is a tertiary-level, stand-alone maternity hospital in Dublin, Ireland, with over 8500 deliveries per year. The Maternal Fetal Medicine and Neonatology departments accept national referrals and have over 1500 admissions to the neonatal unit per year. The study was approved by the National Research Ethics Committee of the National Maternity Hospital and by the Health Products Regulatory Authority in Ireland. Pregnant women who had attained a minimum GA of 31 weeks and up to 40 weeks were recruited to the study. The patients were recruited through the prenatal department in the hospital. If deemed eligible, subjects were approached by the lead study investigator and invited to take part in the study. Inclusion criteria were as follows: age ≥ 18 years with no significant medical history. Participants with a non-smoking status were chosen given the hazards associated with smoking and high flow oxygen [17] and to eliminate any effect that smoking may have on Doppler velocity waveforms [18–20]. Singleton pregnancies with a normally grown fetus (estimated fetal weight ≥ 5th centile and ≤ 95th centile for GA) were included, this was to exclude any effect that growth restriction [21–23] or fetal macrosomia [24, 25] may have on fetal Doppler waveforms. Fetuses ≥31 weeks’ gestation were included as the hyperoxygenation test is known to become responsive after this GA [4]. Those with a GA > 40 weeks were excluded given the potential for advanced GA to affect the acquisition of or the result of various Doppler indices. A window of 31–40 weeks GA was chosen to increase uniformity and to acquire better data. Additional exclusion criteria included known fetal chromosomal abnormality, use of bleomycin or amiodarone, current use of nitrofurantoin or use within the last 7 days as interactions can occur between these drugs and oxygen, use of any pre-existing vasoactive medication that could affect cardiac function, or those unable to provide written informed consent. Limited data exist on the haemodynamic changes in pregnancy in response to MH [26] and for this reason we excluded those with any chronic respiratory disease, congenital heart disease or uncontrolled diabetes. Baseline characteristics of all women were recorded and included maternal age, GA, gravidity, body mass index (BMI) and antepartum haemoglobin.

### Study procedures

**Fig. 1:**
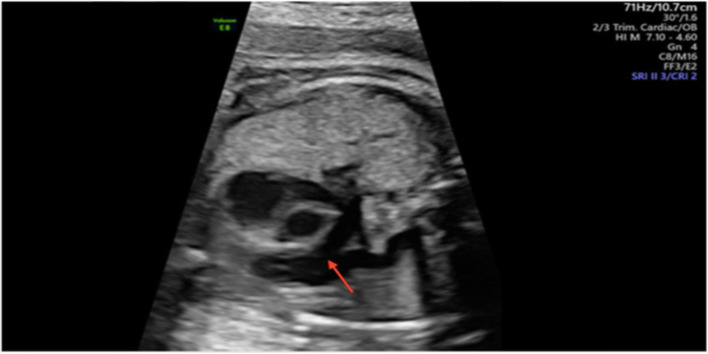
Ultrasound image of the fetal branching pulmonary artery in an extended three vessel view. The red arrow marks the area where the Doppler velocimetry waveform was obtained

**Fig. 2:**
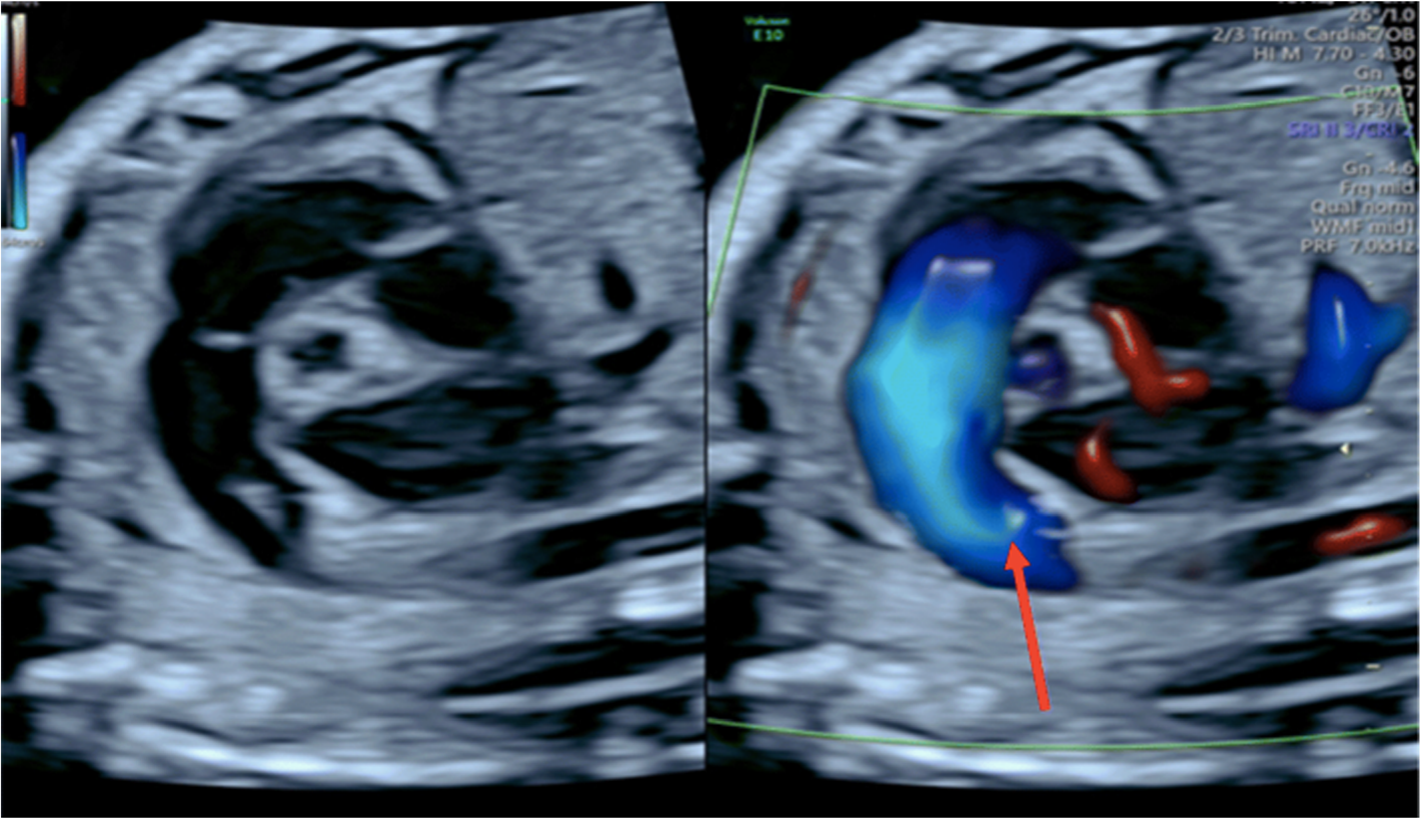
The pulmonary artery as illustrated in this ultrasound image, arises from the anteriorly positioned right ventricle and courses towards the descending aorta. The ductal arch has a nearly perpendicular shape and resembles a hockey stick. The red arrow represents where the ductus arteriosus Doppler waveform was obtained

Image-directed pulsed and colour Doppler equipment (Voluson E8) was used with a 5-MHz sector probe. All Doppler recordings were obtained using the lowest high-pass filter level (100 Hz), and the spatial peak temporal average power output for colour and pulsed Doppler was kept at < 100 mW/cm [27]. An angle of ≤15° between the vessel being studied and the Doppler beam was deemed acceptable and used for analysis. All participants underwent a standard ultrasound examination for estimation of fetal weight, amniotic fluid volume measurements, fetal heart rate and umbilical (UA) and middle cerebral artery (MCA) Doppler assessment. A fetal echocardiogram was performed according to the International Society of Ultrasound in Obstetrics and Gynecology (ISUOG) guidelines for fetal echocardiography [28] on fetuses between 31 and 40 weeks gestation. This involved a sequential segmental analysis of the atria, ventricles, and great arteries and their connections. Specific echocardiographic Doppler studies included those of the distal main PA and ductus arteriosus (DA) (Figs. 1 and 2). The following measurements specific to the PA Doppler waveform were recorded: The peak systolic velocity (PSV), end diastolic velocity (EDV), time-averaged velocity (TAV), pulsatility index (PI), resistance index (RI), ejection time (ET) and acceleration time (AT). The ductus arteriosus waveform was obtained in the traditional longitudinal ductal arch view. Reported values were averaged from three consecutive waveforms. Oxygen was administered to the subjects while in a semi-recumbent position in the hospital ultrasound department, at a rate of 12 L/min for a duration of 10 min via a partial non-rebreather mask. Immediately following MH, a repeat fetal echocardiogram was performed, and all Doppler recordings were repeated. Each fetus served as its own control. Recordings were stored on the ultrasound machine for further analysis and for data safety monitoring purposes. The hyperoxygenation test was considered positive when the PI of the fetal PA decreased by ≥10% from its baseline (responders). Where the fetal PA PI did not decrease by at least 10%, cases were classified as non-responders. The fetal PA Doppler measurements were kept in a specific study file and not in the subject’s hospital file. The calculation of fetal PA reactivity was performed after the ultrasound assessment, when the subject had left the ultrasound room. The subject was not made aware of the prenatal PA reactivity results*.*

A functional neonatal echocardiogram was performed within the first 24 h of life. Neonates were divided into those that responded to MH in utero (responders) and those that did not respond (non-responders). Echocardiography was carried out using a Vivid S6 echocardiography machine and a 7 MHz neonatal probe (GE Medical, Milwaukee, USA). Studies were conducted during a resting state in accordance to recent guidelines and congenital heart disease was excluded during the first scan [29]. Data were stored as raw DICOM images in an archiving system (EchoPac, General Electric, version 112 revision 1.3) and analysis of all the echocardiography parameters was carried out by a single investigator who was blinded to the results of fetal Doppler ultrasounds (N.B). The following echocardiography parameters were also measured using previously described methods [30], left ventricular (LV) length measured at end diastole; mitral value annual diameter; left ventricular output (LVO, ml/kg/min); ejection fraction using Simpson’s Biplane method (EF, %); mitral valve inflow velocities and velocity time index, patent ductus arteriosus (PDA) diameter (mm) measured in 2D at the pulmonary end; diastolic and systolic flow velocity across the PDA; flow pattern across the duct; pulmonary artery acceleration time (PAAT); and right ventricular (RV) end systolic pressure measured using the tricuspid valve regurgitant jet.

### Sample size and statistical analysis

Sample sizes of between 24 and 50 have been recommended variously for pilot studies [31–38]. Following these recommendations, we chose a recruitment sample size of 40–60 which would allow for a moderate dropout rate. A significant dropout rate (e.g. 40%) would reduce the pilot sample size to below a minimum of 24, in which case a possible larger study would be called into question in the first place, having possible external validity issues, pragmatic or ethical concerns. Data analysis was performed using SPSS software (version 24.0; IBM Corporation, Armonk, NY). Continuous data were expressed as means and standard deviations or medians and interquartile ranges as appropriate. Independent data were compared using the independent Student t-test or the Mann-Whitney U test as appropriate. Paired data were compared using the paired Student t-test or the non-parametric equivalent as appropriate. Categorical data were compared using the Chi Square test or the Fisher Exact test. Data were deemed statistically significant at a *p*-value < 0.05.

### Reproducibility bias

Intraobserver and interobserver variability of fetal PA Doppler indices were assessed using a subset of 10 patients. One reader (A.M) repeated measurements at a time temporally remote from the initial assessment. To assess interobserver variability, a second reader (F.B), blinded to the original data, repeated PA Doppler measurements. Intraobserver and interobserver variability was assessed by calculation of mean percent error, defined as the absolute difference between observations divided by the mean of observations and using the intraclass correlation coefficient (ICC) and 95% confidence intervals (95% CI). To assess the repeatability of the measured values, the mean and SD of differences and the repeatability coefficient of the two repeated tests within subjects were calculated. The PA Doppler indices that are reported in the results were taken by a single operator (A.M). The study was not powered as a predictive tool and therefore results may not be reflective of a cause and effect relationship.

## Results

**Fig. 3:**
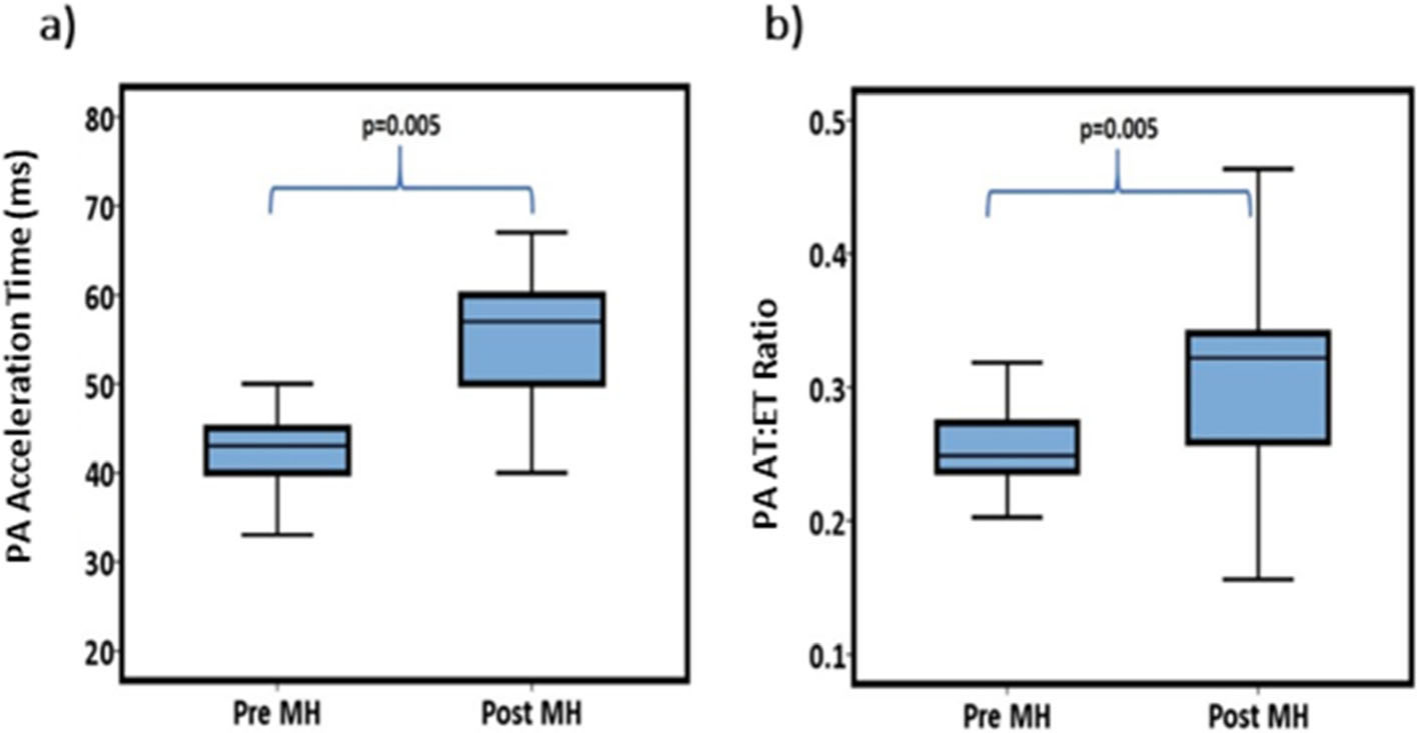
Changes in PA AT: ET following maternal hyperoxygenation. **a** Pulmonary Artery Acceleration Time. **b** Pulmonary Artery AT:ET, Acceleration to Ejection Time Ratio. PA, pulmonary artery; Pre MH, before maternal hyperoxygenation; Post MH, following maternal hyperoxygneation; ms, millisecond

**Fig. 4:**
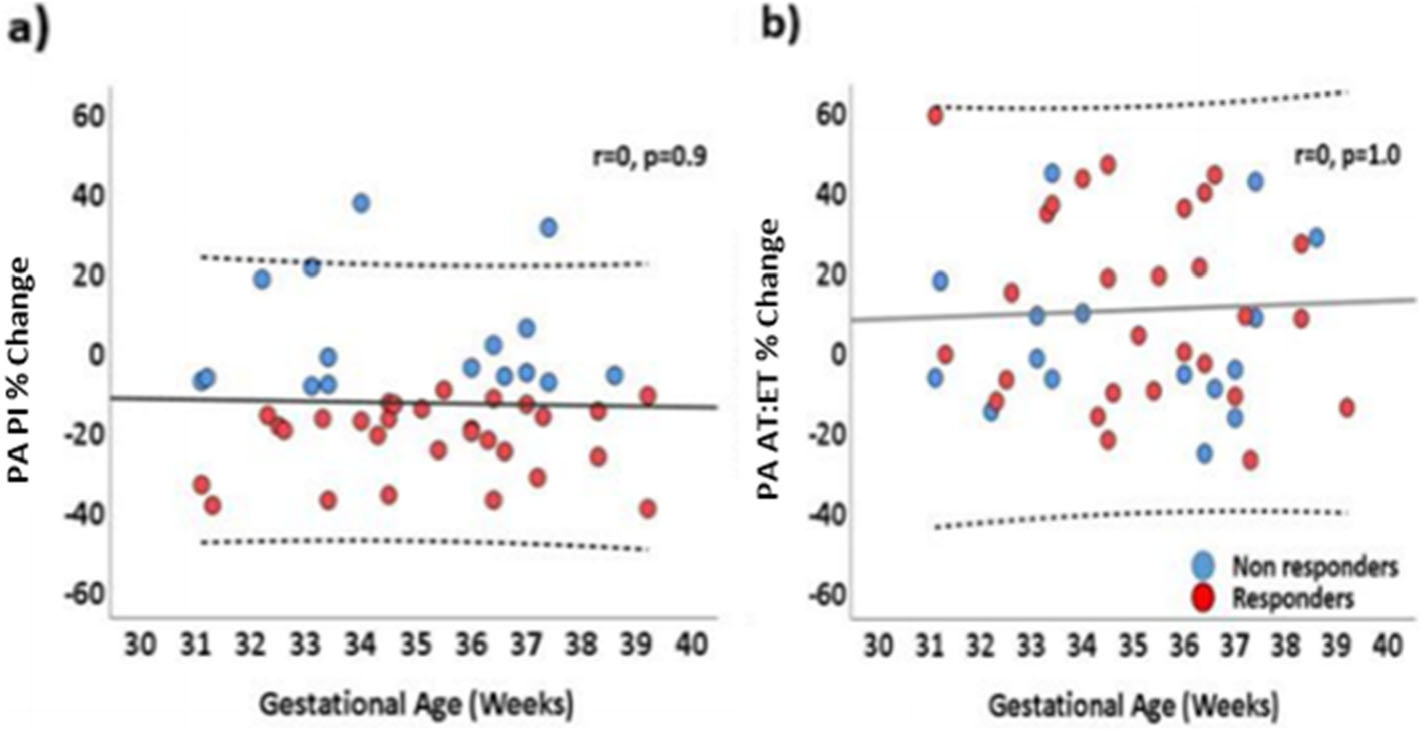
Changes in fetal PA PI and PA AT:ET following maternal hyperoxygenation according to gestational age. **a** Fetal pulmonary artery pulsatility index percentage change in responders and non-responders to maternal hyperoxygenation according to gestational age. **b** Fetal pulmonary artery acceleration to ejection time ratio percentage change in responders and non-responders to maternal hyperoxygenation according to gestational age. R = 0 represents no correlation with gestational age, *p* values not significant

Forty-six women in the third trimester were prospectively recruited to the study. Fetal standard anatomical survey did not reveal any structural abnormalities. The mean maternal age was 32.5 ± 6.2 years. The median GA at the time of the MH test was 35 [IQR 33–37] weeks (Table 1). Each fetus was appropriately grown for GA (all between the 10th and 90th percentile growth curve). The mean estimated fetal weight was 2660 g ± 626 g at the time of the sonographic assessment. In all cases, amniotic fluid volumes based on a single deepest vertical pool were within the normal range (4.8 cm ± 1.8 cm). Successful acquisition of PA Doppler indices were achieved in all participants. A decrease in fetal PA PI was observed following maternal hyperoxygenation, with a median decrease of 21% [9–36] from the baseline. The resistance index of the PA decreased following MH (Table 2). There was an increase in PA AT leading to an increase in PA AT:ET, indicating a fall in pulmonary vascular resistance, following MH (Fig. 3). No changes were observed in the pulsatility indices of the UA or MCA following hyperoxygenation (0.96 to 0.99, *p* = 0.95 and 1.70 to 1.72, *p* = 0.98), respectively. The fetal reactivity, as demonstrated by the PA PI Doppler measurements in responders and non-responders, was not associated with maternal age (31.7 ± 6.4 years vs. 33.8 ± 5.7 years, *p* = 0.29), maternal BMI (29.3 ± 4.8 kg/m^2^ vs. 31.2 ± 6.6 kg/m^2^, p = 0.29) or GA at the time of MH (35.3 ± 2.2 weeks vs. 34.9 ± 2.4 weeks, *p* = 0.57) (Fig. 4).

**Table 1:** Patient demographic data

Basic Demographic data (*N* = 46)	
Age (years)	32.5 ± 6.2
Gestational age (weeks)	35 [33–37]
BMI (kg/m^2^)	30 ± 5.4
Nulliparous	18 (39%)
Caucasian	37 (80%)
Indian/Pakistani/Bangladesi	5 (11%)
African	4 (9%)
Haemoglobin in third trimester (g/dL)	11.9 ± 0.92
Baseline maternal blood pressure (mmHg)	SBP 121 ± 17 DBP 78 ± 10

*BMI* Body Mass Index; *kg/m2* kilograms per metre squared; *g/dL* grams per decilitre; *mmHg* millimetres of mercury; *SBP* systolic blood pressure; *DBP* diastolic blood pressure.

*Values displayed as mean ± SD, median and [IQR] and percentages (%).*

**Table 2:** Doppler changes before and after MH

Doppler measurements	Pre MH	Post MH	***p***-value
PA PSV cm/s	60.62 ± 11.4	59.2 ± 14.1	0.60
PA EDV cm/s	7.75 ± 2.2	8.01 ± 2.1	0.56
PA TAV cm/s	16.18 ± 2.5	18.61 ± 4.9	0.004
PA PI	2.47 ± 0.36	2.08 ± 0.33	0.0001
PA RI	0.86 ± 0.05	0.78 ± 0.09	0.0001
PA TVI cm	5.3 ± 2.1	6.2 ± 1.7	0.026
PA AT ms	43 [40–47]	57 [47–60]	0.005
PA ET ms	177 [163–183]	187 [177–207]	0.005
PA AT:ET	0.25 [0.24–0.28]	0.32 [0.26–0.34]	0.005
DA PSV cm/s	106.8 ± 15.4	109.1 ± 16.7	0.47
DA EDV cm/s	12.4 ± 1.8	11.9 ± 2.2	0.24
DA TAmax cm/s	34.4 ± 7.1	36.4 ± 6.9	0.18
DA PI	2.41 ± 0.36	2.42 ± 0.35	0.89
DA RI	0.87 ± 0.10	0.88 ± 0.12	0.74
UA PSV cm/s	39.8 ± 10	41.6 ± 11	0.28
UA EDV cm/s	16.1 ± 6	16.2 ± 5	0.85
UA TAmax cm/s	26.1 ± 7.5	26.9 ± 7.1	0.60
UA PI	0.96 ± 0.25	0.99 ± 0.23	0.55
UA RI	0.61 ± 0.09	0.62 ± 0.08	0.58
MCA PSV cm/s	33.3 ± 11.5	40.5 ± 17	0.02
MCA EDV cm/s	7.1 ± 3.2	8.7 ± 5.3	0.08
MCA PI	1.70 ± 0.6	1.72 ± 0.7	0.88
MCA RI	0.78 ± 0.09	0.80 ± 0.08	0.26

*Abbreviations: PA* pulmonary artery; *DA* ductus arteriosus, *UA* umbilical artery; *MCA* middle cerebral artery; *PSV* peak systolic velocity; *EDV* end diastolic velocity; *TAV* time-averaged velocity; *PI* pulsatility index; *RI* resistance index; *TVI* time velocity integral; *TAmax* time-averaged maximum velocity; *AT* acceleration time; *ET* ejection time; *AT:ET* acceleration time to ejection time ratio; *cm* centimetres; *cm/s* centimetres per second; *ms* millisecond. *MH* maternal hyperoxygenation. Values displayed as means ± SD or median and [IQR]

There were no significant changes in the peak systolic, end-diastolic, mean velocities or resistance indices across the DA following MH. Fetal heart rate did not change significantly in response to MH (140 ± 12 bpm versus 136.8 ± 8 bpm, *p* = 0.08). There was a significant increase in MCA blood flow assessed using PSV, but not in MCA resistance indices. Intraobserver and interobserver variability of PA flow velocities (PI, RI) were low, with mean percent errors ranging from 4 to 6%. High levels of ICC (95% CI) were demonstrated (Supplementary Table 1).

The Caesarean delivery rate in this cohort was 54.3% (25/46). The higher caesarean section rate and lower gestational age at delivery in the cohort is partly accounted for by the fact that twelve subjects (26%, *n* = 12/46) were long stay inpatients in the hospital due to a diagnosis of placenta praevia, all of these patients underwent caesarean section at less than 38 weeks GA. A physical examination at birth was recorded as normal for all newborn subjects. A neonatal echocardiogram was performed on 74% (*n* = 34/46) of recruited cases. Echocardiography was carried out at a median of 18 h [IQR 12–26] of life. Of the 12 neonates that did not undergo an echocardiogram, one had a fragile skin condition at birth and the test was deferred, one declined participation and ten infants were discharged home from the hospital before one of the study investigators could perform the echocardiogram. No neonate was diagnosed with persistent pulmonary hypertension. There was no difference in mean GA at delivery or in mean birthweight between the two groups (Table 3). Fetuses that responded to MH (a decrease PA PI > 10% from baseline) were more likely to have a higher LVO (135 ± 25 mL/kg/min verses 111 ± 21 mL/Kg/min, p = < 0.01) and EF (54 ± 9% versus 47 ± 7%, *p* = 0.03) within the first 24 h of life. These findings were not dependent on LV length or MV annular diameter but were related to an increase in MV inflow demonstrated by an increase in the MV velocity time integral (8.6 ± 1.6 versus 7.4 ± 0.9, *p* = 0.01). There were no differences in nPAAT, RV end systolic pressures, or PDA characteristics between the two groups (Table 3).

**Table 3:** Neonatal Data

	Responders (***n*** = 19)	Non-responders (***n*** = 15)	***p***-value
Gestation (weeks)	37.4 ± 1.9	38.0 ± 1.6	0.32
Birthweight (grams)	3190 ± 611	3234 ± 409	0.80
LV length (mm)	28 ± 4	28 ± 4	0.88
Mitral Valve Annular diameter (mm)	8.6 ± 1.4	9.4 ± 0.9	0.08
Ejection Fraction (%)	54 ± 9	47 ± 7	0.03
Left Ventricular Output (mL/Kg/min)	135 ± 25	111 ± 21	< 0.01
Mitral Valve Inflow VTI	8.6 ± 1.6	7.4 ± 0.9	0.01
nPAAT (msec)	78.6 ± 26	78.3 ± 21	0.53
RV end systolic pressure (mmHg)	18.6 ± 12.2	17.4 ± 6.9	0.81
PDA characteristics (mm)	2.6 ± 1.3	2.4 ± 1.5	0.74

Abbreviations: *LV* left ventricle; *VTI* velocity time integral, *nPAAT* neonatal pulmonary artery acceleration time; *RV* right ventricle, *PDA* patent ductus arteriosus; *mm* millimetres; *%* percentage; *mL/Kg/min* millilitres per kilogram per minute. All values reported are means ± SD

The effect of MH on neonatal LVO was not dependent on mode of delivery (standardised β = 0.13, *p* = 0.48), GA at delivery (standardised β = 0.05, *p* = 0.80) or infant heart rate (standardised β = − 0.05, *p* = 0.77) at the time of the neonatal echocardiogram. However, neonatal LVO was dependent on the prenatal response to MH (standardised β = 0.51, *p* < 0.01). The neonatal EF was also not dependent on the mode of delivery (standardised β = − 0.17, *p* = 0.29) or GA at delivery (standardised β = 0.02, *p* = 0.89) but was dependent on the prenatal response to MH (standardised β = 0.34, *p* = 0.04) and on infant heart rate (standardised β = − 0.41, *p* = 0.02), (Table 4).

**Table 4:** The Independent Effect of the response to Maternal Hyperoxygenation on Neonatal Left Ventricular Output (Model 1) and Ejection Fraction (Model 2)

Dependent Variable	Model 1: LVO		Model 2: EF	
***Predictor Variables***	Standardised β	p	Standardised β	p
*MH Response*	0.51	< 0.01	0.34	0.04
*Mode of Delivery*	0.13	0.48	−0.17	0.29
*Gestation at Delivery*	0.05	0.80	0.02	0.89
*Infant Heart Rate*	−0.05	0.77	−0.41	0.02

*Linear Regression Analysis assessing the independent effect of maternal hyperoxygenation response on neonatal left ventricular output (Model 1) and ejection fraction (Model 2).*

*Abbreviations: LVO, left ventricular output; EF, ejection fraction; MH, maternal hyperoxygenation*

## Discussion

We have demonstrated that maternal hyperoxygenation during the third trimester is associated with significant changes in fetal Doppler waveforms, characterised by a decrease in the PA PI and PA RI and an increase in the PA AT:ET. These changes did not occur at the expense of ductal constriction. These findings were not related to a change in the resistance indices of the uteroplacental circulation. Fetuses that were classified as responders to MH were more likely to have a higher left ventricular output and ejection fraction during the early neonatal period. The neonatal echocardiographic changes were due to an increase in mitral flow velocity (likely reflecting increased pulmonary blood flow) and not related to an increase in left ventricular size.

In the human fetus, blood flow velocity waveforms can be recorded from the right and left pulmonary arteries or from peripheral vessels within the lung. Analysis of the waveforms using ultrasound Doppler can be used to study the normal development of fetal lung circulation [39]. Doppler examination of blood flow in the main stem of the fetal pulmonary arteries is feasible and increases our insight into the lung perfusion of the human fetus. Mean decreases in PA PI following MH of between 18.0 and 21.2% have been previously described in normal fetuses [4]. A cut off level of ≥20% decrease in the PA PI from the baseline have been studied and deemed to demonstrate pulmonary reactivity [4, 40]. However, there remains large individual variability [41, 42]. This variability can also be identified when the same fetus is serially assessed as gestation advances. In one study of normal fetuses, nearly one-third had a decrease in PA PI that was less than 20% after an initial positive oxygenation test earlier in gestation [13]. Values for reactivity in our study accounted for this variability and were based on a previous study where a decrease in PA PI of ≥10% from the baseline level was used to characterise a reactive test or positive responder [43].

The increase in fetal pulmonary blood flow following MH results in increased venous return to the left heart and this response increases with gestational age [44]. Studies indicate that a lack of vasoreactivity in response to MH may serve as a useful clinical tool in predicting lethal pulmonary hypoplasia in at-risk fetuses [40, 43]. The reactivity of the fetal pulmonary arteries to hyperoxygenation and its effect on neonatal cardiac function has not been established. Our study confirms previous findings of a significant decrease in fetal PA PI following MH [4, 40, 45]. Doppler echocardiography derived PA AT correlates with invasively derived PA pressures and PVR in children [46]. We have also demonstrated an increase in fetal PA AT:ET following MH, indicating a fall in PVR. These data support previous reports in animal and human studies that a decrease in the pulmonary vascular impedance during MH is not caused by a constriction of the DA [4, 27, 47].

The effects of MH on fetal haemodynamic indices are limited, owing to a relatively high fetal umbilical vein oxygen saturation during normoxia, due to the high oxygen affinity of fetal haemoglobin [48]. Maternal hyperoxygenation has been shown to increase fetal partial pressures of oxygen in the umbilical artery and vein, as well as umbilical arterial oxygen saturations [49, 50]. Our findings are in keeping with other studies where maternal hyperoxia induced no changes to umbilical artery Doppler resistance indices [12, 51].

The fetal ability to modify cardiac output is limited [7, 52]. Increases in preload, prenatally, have a minimal impact on fetal cardiac output, as the fetal heart is functioning at the peak of the Frank-Starling ventricular function curve [7]. There is a doubling of LVO in normal fetuses, within the first hour of life [53]. Increased pulmonary blood flow leads to a steady fall in PVR due to increased nitric oxide production. Within the first 24 h of life pulmonary arterial pressure has reached half that of systemic arterial pressure [6]. Our study demonstrated evidence of improved fetal MCA peak systolic velocity parameters in response to MH, most likely due to the positive impact of improved pulmonary venous return on left ventricular preload and fetal left ventricular cardiac output.

All fetuses were normally grown and without structural abnormality at the time of recruitment. However, there were significant postnatal differences between those that responded to MH and those that did not. Those that responded appropriately to MH, had signs of improved neonatal cardiac output with increased LV ejection fractions compared to poor responders as increased mitral valve flow velocities were observed in the responder group. It is likely that increased pulmonary blood flow due to an optimal postnatal pulmonary vasodilatation in the cohort observed to have the best response to MH prenatally was the basis of the measured values.

The most immediate and adverse adaptation of the fetal to neonatal transition is the persistence of high PVR [54]. This can result in continued right to left or bidirectional shunting across the DA and/or foramen ovale leading to reduced pulmonary blood flow. Reduced pulmonary blood flow generates decreased pulmonary venous return and accordingly decreased LV preload and low LVO [55]. If this cycle continues, decreased organ perfusion will ensue, resulting in an increase in lactate, acidosis and hypoxia which are potent pulmonary vasoconstrictors and ultimately can lead to the development of pulmonary hypertension [56, 57]. The only predictor of a reduced neonatal LVO in our study was the fetal response to MH in utero. The LVO was not dependent on mode of delivery or GA at delivery and was not dependent on fetal heart rate. This interesting observation suggests that the neonatal compromise due to caesarean delivery is primarily a respiratory problem and that there is a cohort of infants who may be identified by MH prenatally, who would be predicted to have an increased vulnerability to deal with neonatal illness, due to a limited ability to achieve the pulmonary vasodilatation necessary to optimise LV preload and therefore achieve the increase in cardiac output demanded of the neonatal circulation.

### Strengths and limitations

To our knowledge, this is the first study to compare the effects of MH with neonatal echocardiography evaluation, in normal pregnancies. All fetal Doppler measurements included in the study results were performed by a single operator (A.M). All neonatal echocardiography measurements were carried out by a single investigator who was blinded to the results of the fetal Doppler assessments (N.B).

We acknowledge the small sample size in our study. This warrants further exploration in a larger cohort to establish the ability of the fetal response to MH to predict postnatal adaptation of the pulmonary vascular circulation during the transition to neonatal life.

## Conclusion

We have demonstrated that the extent of the response to maternal hyperoxygenation during fetal life may predict the rate of transition to the neonatal circulation, during the early neonatal period. Our study suggests that the prenatal hyperoxygenation test may offer the potential to predict an optimal adaptation to postnatal life, as evidenced by rapid postnatal reduction in pulmonary vascular resistance increasing measured cardiac output. The clinical implications of these findings require further investigation particularly in the context of pathologic conditions such as lung hypoplasia and congenital cardiac disease.

## Supplementary Information

**Additional file 1: Supplementary Table S1.** Intraobserver and Interobserver Repeatability of Pulmonary Artery Doppler Measurements

## Data Availability

The detailed datasets used and analysed during the study are available from the corresponding author on reasonable request.

## Abbreviations

PI: Pulsatility index
RI: Resistance index
AT: Acceleration time
ET: Ejection time
AT:ET: Acceleration to ejection time ratio
PA: Pulmonary artery
MH: Maternal hyperoxygenation
EF: Ejection fraction
LVO: left ventricular output
nPAAT: Neonatal pulmonary artery acceleration time
MV: Mitral valve
PVR: Pulmonary vascular resistance
GA: Gestational age
BMI: Body mass index
UA: Umbilical artery
MCA: Middle cerebral artery
DA: Ductus arteriosus
PSV: Peak systolic velocity
EDV: End diastolic velocity
TAV: Time-averaged velocity
LV: Left ventricular
PDA: Patent ductus arteriosus
RV: Right ventricular
ICC: Intraclass correlation coefficient
TVI: Time velocity integral
TAmax: Time-averaged maximum velocity
