# Supplementary material for: Prenatal prediction of neonatal haemodynamic adaptation after maternal hyperoxygenation
Source: BMC Pregnancy Childbirth. 2020 Nov 19;20:706. doi: 10.1186/s12884-020-03403-y (PMC7678134; doi:10.1186/s12884-020-03403-y)
Supplement: Supplementary file 1 — Additional file 1: Supplementary Table S1. Intraobserver and Interobserver Repeatability of Pulmonary Artery Doppler Measurements [file 12884_2020_3403_MOESM1_ESM.docx]

**Supplementary Table 1:**

**Intraobserver and Interobserver Repeatability of Pulmonary Artery Doppler Measurements**

| **Doppler Parameter** | **Mean ± SD** | **RC** | **CV (%)** | 1. **ICC (95% CI)** |
| --- | --- | --- | --- | --- |
| 1. **Intraobserver** | | | | |
| 1. PA PI (A.M) 2. R | 1. 2.33 ± 0.14 2. 2.32 ± 0.12 | 1. 0.28 | 1. 9 | 1. 0.99 (0.96-0.99) |
| 1. PA RI (A.M) 2. R | 1. 0.87 ± 0.03 2. 0.87 ± 0.04 | 1. 0.59 | 1. 4 | 1. 0.97 (0.88-0.99) |

|  | 1. **Mean ± SD** | 1. **Difference (95% CI)** | 1. **ICC (95% CI)** |
| --- | --- | --- | --- |
| 1. **Interobserver** | | | |
| 1. PA PI (A.M) 2. PA PI (F.B) 3. R | 1. 2.35 ± 0.14 2. 2.36 ± 0.15 | 1. 0.046 (-0.075 - 0.11) | 1. 0.98 (0.90-0.99) |
| PA RI (A.M)  PA RI (F.B)  R | 1. 0.86 ± 0.03 2. 0.86 ± 0.04 | 1. 0.12 (-0.024-.024) | 1. 0.93 (0.71-0.98) |

*Abbreviations: PA, pulmonary artery; PI, pulsatility index; RI, resistance index; SD, standard deviation; RC, repeatability coefficient; CV, coefficient of variation, ICC, intraclass correlation coefficient. The repeatability coefficient has been defined as 1.96 times the SD of differences between repeated measurements.* *the coefficient of variation (CV) was calculated from the two repeated tests. The CV was defined as the SD of the error in a single test and expressed as a percentage of the mean PA PI. A.M and F.B denote the study investigator that obtained the Doppler measurement. R denotes the repeatability measurements.*
